# Ultrasound-Based Scoring System for Indication of Pyeloplasty in Patients With UPJO-Like Hydronephrosis

**DOI:** 10.3389/fped.2020.00353

**Published:** 2020-07-02

**Authors:** Bruce Li, Melissa McGrath, Forough Farrokhyar, Luis H. Braga

**Affiliations:** ^1^Michael G. DeGroote School of Medicine, McMaster University, Hamilton, ON, Canada; ^2^McMaster Pediatric Surgical Research Collaborative, McMaster University, Hamilton, ON, Canada; ^3^Division of Urology, McMaster University, Hamilton, ON, Canada; ^4^McMaster Children's Hospital Foundation, Hamilton, ON, Canada; ^5^Department of Health Research, Methods, Evidence & Impact, McMaster University, Hamilton, ON, Canada

**Keywords:** ureteropelvic junction obstruction, prenatal hydronephrosis, pyeloplasty, classification, ultrasonography

## Abstract

**Background:** Previous scoring systems have used renal scan parameters to assess severity of ureteropelvic junction obstruction-like hydronephrosis (UPJO-like HN), however this information is not always reliable due to protocol variation across centers and renogram limitations. Therefore, we sought to evaluate the Pyeloplasty Prediction Score (PPS), which utilizes only baseline ultrasound measurements to predict the likelihood of pyeloplasty in infants with UPJO-like.

**Methods:** PPS was developed using three ultrasound parameters, Society of Fetal Urology (SFU) grade, transverse anteroposterior (APD), and the absolute percentage difference of ipsilateral and contralateral renal lengths at baseline. PPS was evaluated using prospectively collected prenatal hydronephrosis data (*n* = 928) of patients with UPJO-HN. Children with vesicoureteral reflux. primary megaureter, other associated anomalies, bilateral HN and <3 months of follow-up were excluded. Scores were analyzed regarding its usefulness in predicting which patients would be more likely to undergo pyeloplasty. Sensitivity, specificity, likelihood ratios (LR) and receiver operating characteristic (ROC) curve were determined.

**Results:** Of 353 patients, 275 (78%) were male, 268 (76%) had left UPJO-like HN, and 81 (23%) had a pyeloplasty. The median age at baseline was 3 months (IQR 1–5). The PPS system was highly accurate in distinguishing patients who underwent pyeloplasty using baseline ultrasound measurements (AUC: 0.902). PPS of 7 and 8 were found to have a sensitivity of 85 and 78%, and specificity of 81 and 90%, respectively. PPS of 8 was associated with a LR of 7.8, indicating that these patients were eight times more likely to undergo pyeloplasty.

**Conclusion:** Overall, PPS could detect patients more likely to undergo pyeloplasty using baseline ultrasound measurements. Those with a PPS of eight or higher were eight times more likely to undergo pyeloplasty.

## Introduction

Prenatal hydronephrosis is one of the most commonly detected ultrasound findings, affecting 1–5% of pregnancies, and is usually detected during the third trimester as an incidental finding (1). Ureteropelvic junction obstruction-like hydronephrosis (UPJO-like HN) is one of the most common congenital causes of prenatal hydronephrosis (1). If left untreated, the severe hydronephrosis (HN) due to obstruction can lead to a clinical symptoms such as urinary tract infections, hematuria, progressive deterioration of renal function, and permanent kidney damage (1–3). Thus, early detection and surgical intervention of UPJO cases provides benefits by reducing the length of time the kidney is obstructed. However, a large proportion of UPJO-like [isolated hydronephrosis (pelvic distension) with or without dilated calyces] cases are benign in nature and spontaneously resolve. Therefore, the challenge with UPJO-like patients is identifying those that warrant further testing and would benefit from intervention in a timely manner to reduce associated morbidities.

Scoring systems have been developed to be utilized as an adjunctive tool to help predict those patients in need of pyeloplasty. Many of these scoring systems rely on ultrasound measurements of the afflicted kidney and on diuretic renogram findings. Nevertheless, nuclear studies pose an issue to the external validity of these scoring systems, as their protocols vary significantly across different centers (4). Consequently, interpretation of the drainage patterns, renogram curves and T1/2 times can be subjective and unreliable.

The primary objective of this study was to create a scoring system, the pyeloplasty prediction score (PPS), based on baseline ultrasound findings only, and evaluate its utility in predicting pyeloplasty in infants with UPJO-like. We hypothesized that the proposed scoring system could discriminate those who will resolve spontaneously from those who will end up having surgical intervention.

## Materials and Methods

After obtaining Research Ethics Board approval (13-62D), we reviewed our prospectively collected prenatal hydronephrosis database (*n* = 928) from a tertiary pediatric hospital and identified those who were diagnosed with UPJO-like hydronephrosis between 2008 and 2019. We excluded infants with vesicoureteral reflux, primary megaureter (hydroureteronephrosis), duplication anomalies, bilateral cases, other genitourinary anomalies (Prune-Belly, posterior urethral valves, horseshoe kidneys, neurogenic bladder, multicystic dysplastic kidney), and those with <3 months of follow-up.

### Calculation of the Pyeloplasty Prediction Score

For each case, characteristics were collected, and only baseline (initial visit) ultrasound measurements were analyzed. The PPS scoring system was then retrospectively applied to each included case. Ultrasound measurements were conducted following institutional protocol, such as no pretest patient hydration to minimize measurement bias, with the patient in supine position, measurement pre- and post-void to confirm an empty bladder, using the same two ultrasound machines, by two technicians specialized in pediatric renal bladder ultrasound, who were specifically assigned to the Pediatric Urology Service.

The clinical outcome was resolution of HN or surgical intervention with a pyeloplasty. HN resolution was defined as two consecutive ultrasounds showing either Society for Fetal Urology (SFU) grade 1 or less, or renal pelvis anteroposterior diameter (APD) of 10 mm or less (5). Indications for pyeloplasty were based on the following protocol: **1-**Worsening of hydronephrosis, characterized by increase in the transverse APD of the renal pelvis with or without change (increase) of SFU grade on repeat ultrasounds; or **2-**Deterioration of differential renal function (DRF) >10% on repeated renal scans; or **3-**Initial renal function <40% associated with an obstructive (ascending) curve on renogram; or **4-**Worsening of hydronephrosis associated with a T1/2 time >30 min, or **5-**Development of symptoms (sepsis, febrile urinary tract infections, stones).

**Figure 1:**
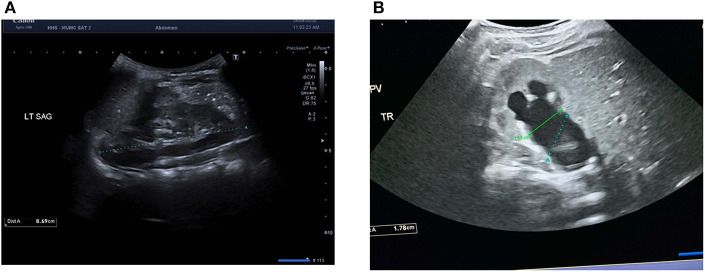
Example measurements of ultrasound renal parameters for the Pyeloplasty Prediction Score in **(A)** longitudinal view of the left kidney with electronic calipers measuring renal length as the maximal distance between the upper and lower poles and **(B)** transverse view of the right kidney with green electronic calipers measuring true anteroposterior diameter (APD) as the distance between the parenchymal lips at the renal hilum in the mid-section. Blue electronic calipers represent the incorrect method of measuring APD as the calipers are not aligned to the parenchymal lips.

PPS was based on three widely used ultrasound variables at baseline: SFU grade, transverse APD, and absolute percentage difference in renal length. APD was calculated in the transverse view, by measuring the distance between the parenchymal lips at the renal hilum in the mid-section. The extra-renal and intra-renal measurements for APD were taken and the larger of the two was recorded in the prospective database to be used in the PPS calculation. Renal length was measured in ipsilateral and contralateral kidneys in the longitudinal view, such that the distance between the most distant points of the upper and lower poles was captured. Figures 1A,B demonstrate the technique for measuring renal length and APD using electronic calipers, respectively. Each of these variables were assigned a value out of four, with zero being normal variant or least severe and four being the most severe; thus, making the PPS score total range from 0 to 12.

The SFU grading system ranges from normal, 1, 2, 3, 4 which corresponded to a score of 0, 1, 2, 3, 4, respectively (6).

The APD measurement was grouped as <5, 5–10, 11–15, 16–19, ≥20 mm corresponding to scores of 0, 1, 2, 3, 4, respectively. The APD category values were established based on current evidence that generally, the larger the APD, the greater the risk of obstructive uropathy (7–9), and thus a greater likelihood of surgical intervention (8, 10–12). An APD <5 mm is not considered as HN, which is why a score of 0 was assigned. A post-natal APD value <10 mm is considered as physiologic HN and APD values from 10 to 15 mm are associated with low risk of obstructive uropathy, which both correspond to the Urinary Tract Dilation (UTD) Classification System's P1 designation (13). The P1 designation is the lowest risk stratum in the UTD post-natal classification, which is why those two APD categories been assigned lower severity scores as 1 and 2, respectively. An APD value of 16 or greater was found by Dias et al. to have the best diagnostic odds ratio to identify infants who had pyeloplasty performed, which corresponds to the category of 16–19 mm (10). Multiple literature sources vary in terms of what APD cutoff value has the greatest likelihood of surgery, but are generally consistent in that an APD of at least 20 mm or greater is associated with the highest likelihood of pyeloplasty, which is why it was designated the greatest score of 4 (14, 15).

Absolute percentage difference between renal lengths was grouped as <5% (error variation), 5–10, 11–15, 16–19, ≥20% corresponding to scores of 0, 1, 2, 3, 4, respectively. The absolute percentage difference was taken by the following equation:

$$\begin{array}{l} \left[100\%\times \frac{\left(\begin{array}{c} Ipsilateral\;Renal\\ Length \end{array}-\begin{array}{c} Contralateral\;Renal\\ Length \end{array}\right)}{Ipsilateral\;Renal\;Length}\right]. \end{array}$$

The three scores were then summed for a total score out of 12. The details of scoring criteria are described in Table 1.

**Table 1:** The Pyeloplasty Prediction Score is based on three parameters: society of fetal urology (SFU) grade of the ultrasound, transverse anteroposterior diameter (APD) measurement, and the absolute percentage difference between the lengths of the ipsilateral and contralateral kidneys.

**A**.	**SFU grading of affected kidney on ultrasound**
0	Normal
1	SFU Grade 1
2	SFU Grade 2
3	SFU Grade 3
4	SFU Grade 4
**B**.	**APD measurement of affected kidney on ultrasound**
0	<5 mm
1	5–10 mm
2	11–15 mm
3	16–19 mm
4	≥20 mm
**C**.	**Absolute percentage difference between the ipsilateral and contralateral renal lengths |[100%** * **(Ipsilateral Renal Length- Contralateral Renal Length)/Ipsilateral Renal Length]|**
0	<5%
1	5%–10%
2	11%–15%
3	16%–19%
4	≥20%
PPS = A + B + C

*Each parameter is assigned a score from 0 to 4, 0 being least severe and 4 being most*.

The PPS system was analyzed for its usefulness in predicting which patients were more likely to undergo pyeloplasty. A trial of various cut-points was done to establish an optimal threshold that would maximize sensitivity and specificity of the scoring system. Sensitivity, specificity, likelihood ratios (LR) with their corresponding 95% confidence intervals (CI) and a receiver-operating characteristic (ROC) curve were determined. A *p*-value equal to or < 0.05 was considered statistically significant. SPSS version 26 (www.ibm.com) were used for analysis.

## Results

Overall, from 928 prenatal HN patients in our database, a total of 353 with UPJO-like (isolated HN) qualified for analysis based on inclusion and exclusion criteria. Of the 353 included infants, 275 (78%) were male and 268 had HN on the left side (76%). The median age of the cohort at baseline (initial visit) was 3 months (IQR 1–5 months). In 81 of the 353 patients (23%), kidneys were considered obstructed based on our criteria (previously stated in the methods section), and a pyeloplasty was performed.

**Figure 2:**
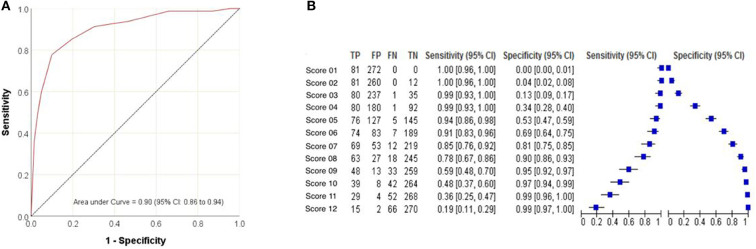
Predictive ability of the Pyeloplasty Prediction Score modeled by **(A)** receiver operating characteristic curve for pyeloplasty and **(B)** sensitivity and specificity at various score cut-points.

The area under the ROC curve (AUC) was 0.902, demonstrating the accuracy of the PPS score in identifying patients more likely to undergo a pyeloplasty (Figure 2A). The PPS could result in a score of 1 to 12, through testing of various modeling scenarios, a score of 7–8 was found to be the optimal cut-off point, with the highest levels of sensitivity and specificity for discriminating patients that would likely be candidates for a pyeloplasty. The sensitivities of a PPS score of 7 and 8 were found to be 85 and 78%, respectively (Figure 2B). The specificities of a PPS score of 7 and 8 were found to be 81 and 90%, respectively (Figure 2B).

**Figure 3:**
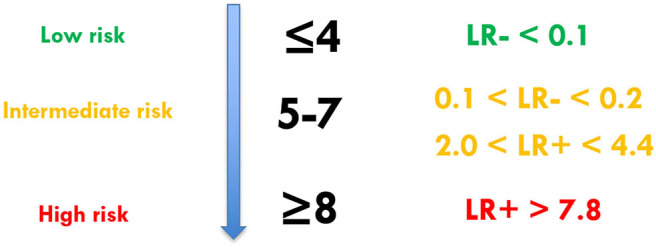
Pyeloplasty Prediction Score stratification according to likelihood ratios.

The LRs of the PPS score range (1–12) increased progressively as the score increased, as expected. The optimal cut-point score of 8 was found to have a LR of 7.8 (Figure 3). Based on LR values, we stratified the patients into three risk categories, according to the likelihood of undergoing pyeloplasty (Figure 3).

## Discussion

The present study involved the development and analysis of a prediction scoring system for pyeloplasty in UPJO-like HN using only baseline ultrasound characteristics. Our findings show that PPS was highly accurate in distinguishing patients who ended up having a pyeloplasty from those managed non-surgically. Based on our findings, the optimal cut-off point where pediatric urologists could consider indicating a pyeloplasty should be a PPS ≥8, provided they followed the same pyeloplasty indications, as outline in our protocol. Only two patients were recorded as false negatives, such that the PPS score was below eight at baseline, yet eventually had pyeloplasty. These two cases initially presented to office with very mild hydronephrosis but over repeated follow-up found worsening of the condition. As previously described, one of the indications for pyeloplasty at our institution is worsening of hydronephrosis by APD or SFU grade, which is why these two patients qualified for surgical intervention. With respect to false positives, there were no patients that scored above 8 and did not have surgical intervention. Based on the sensitivity and specificity calculations, clinicians can expect a 90% probability that those with a score ≥ 8 will end up having a pyeloplasty in the future. The LR indicated that patients with a PPS ≥ 8 were eight times more likely to have surgery vs. no surgery.

### Indications for Pyeloplasty

The indications for pyeloplasty at our center are consistent with what has been previously published in the literature. Within the entire prenatal hydronephrosis database of 928 patients, there were 353 cases of UPJO-like HN which were followed prospectively. Of those 353 patients, only 81 had surgical intervention, which translates into a pyeloplasty rate of 23%. Dhillon et al., one of the first groups to introduce the concept of non-surgical management for UPJO-like HN, had highlighted that approximately one-third of the infants in their series ended up having surgical intervention, which is similar to our figures (15).

Pyeloplasty indications have been well-established in the main urological textbooks. According to Campbell-Walsh 12th edition textbook, widely accepted indications for pyeloplasty include “increasing APD on ultrasonography, low or decreasing DRF, breakthrough infections while on prophylactic antibiotics, or symptomatic hydronephrosis in older infants and children” (16).

Nevertheless, controversy surrounding some of these indications due to inherent subjectivity still exists. Low or decreasing differential renal function does not specify an actual value for decreased function or decreasing function, thus how low or how much has decreased to indicate need for pyeloplasty is subjective to some degree. Some authors may consider <40% DRF as a cut-off (17) while others may push it even lower to <35% (18). Similarly, this subjectivity issue arises with increase in the APD of renal pelvis. At what APD value and at what rate of increase does pyeloplasty outweigh non-surgical management? Again, these values vary from surgeon-to-surgeon and are the subject of many debates within pediatric urology.

Historically, decreased or decreasing renal function as an indication for pyeloplasty had been controversial (17). Waiting until function has dropped and then performing surgery with the hopes to regain what has already been lost seems to be contradictory to the philosophy in pediatrics of maximizing a child's potential (19). While this view does not convey the thought that surgery should be performed on every child, this does highlight the need for a more advanced measure for screening patients that would benefit significantly from early surgical intervention rather than observation.

### Early Intervention Compared to Non-surgical Management

Argument against early intervention of UPJO-like HN consists of evidence demonstrating that most cases of UPJO-like HN are clinically benign and will self-resolve. Koff followed neonates with suspected UPJO-like HN (regardless of degree of HN, shape of diuretic renogram curve, or initial degree of functional impairment) and showed that only 7% eventually had pyeloplasty performed for obstruction, suggesting that due to diagnostic inaccuracy and low risk of developing obstructive injury, many newborn kidneys with HN may rapidly improve without intervention (20, 21). This was further validated by Onen et al. who followed 19 newborns (38 kidneys) with primary SFU grade 3 to 4 bilateral HN for a mean of 54 months. Overall, 25 hydronephrotic kidneys (65%) resolved spontaneously, with renal dilatation and function improving over time in most kidneys (22). Furthermore, Braga et al. analyzed a cohort of 501 UPJO-like HN patients with all SFU grades and observed that 68% of those with grades 3 and 4 HN resolved with non-surgical management over 48 months of follow-up (23). This rate compares well to a recent study from a center known for its conservative approach regarding pyeloplasty indications. They reported a pyeloplasty rate of 38% in 64 patients with grades 3/4 UPJO-like HN at a median age of 21 months (24).

In contrast, benefit of early pyeloplasty in UPJO-like HN has been vastly reported in the literature. With respect to renal function, Babu et al. compared children with UPJO-like HN and SFU grade 3 or 4 who had pyeloplasty done at a mean age of 2.8 vs. 12.5 months. They found that at 1 year follow-up, the early group had a significant improvement of split DRF while the delayed group generally had a marginal loss in function (25). Tabari et al. analyzed functional and anatomic indices (cortical thickness, polar length, SFU grade) in patients with early surgical pyeloplasty compared to those with non-surgical management. The early surgical group noted a faster return to anatomical and functional baseline parameters, whereas the non-surgical group had a significant deterioration in function compared to baseline (26). Thus, it is clinically essential to be able to identify those patients with UPJO-like HN that would benefit most from early pyeloplasty, which is exactly what the PPS was intended to do.

### Limitations of Other Scoring Systems

Other scoring systems, such as the Hydronephrosis Severity Score (HSS) developed by Babu et al. attempted to predict pyeloplasty using ultrasound and diuretic renogram results (27). The main limitation of the HSS is that it relies greatly on the diuretic renogram and the interpretation of its curve, all factors which are heavily exam and operator dependent. Confounders such as time of furosemide dose (F + 20, F – 15, F – 0), bladder catheterization or no catherization, oral or intravenous hydration, DRF of the affected kidney, and conjugate views, all may influence the results of the scan (4, 28–31).

Bladder distension and elevated bladder pressures can restrict the upper urinary tract's ability to drain and can prolong the excretory phase, which is difficult to control without bladder catheterization. Patient position has also been demonstrated to affect urine flow, such that when the patient is supine the urine flow can resemble obstruction whereas upright gravity-assisted position can increase flow significantly (29). Timing of furosemide administration is controversial. Earlier furosemide administration (F + 0, F – 15) urine flow is dramatically increased and can increase the specificity by decreasing the false-positive rate but also results in underestimation of renal function due to acceleration of renal transit (30, 32). Later administration of furosemide (F + 20) allows the examiner to compare the drainage curve before and after furosemide to directly observe the modifications to excretion by diuretic. However, prolongation of the excretory phase does run the increased risk of false-positive findings of obstruction (28). It is not difficult to imagine that even with just the variability of one of these three factors, how many protocol variations can be expected across different centers. This will lead to inconsistencies when interpreting study results involving different protocols and radiotracers.

### Pyeloplasty Prediction Score Parameters

Therefore, the concept of creating a score relying exclusively on ultrasound parameters was attractive because of its reproducibility. SFU grade, APD and renal length discrepancy measurements were chosen as the components of the PPS system because each one of them has been shown to be significantly associated with obstruction/pyeloplasty, as previously reported. Increasing severity of SFU grade, specifically SFU grades 3 and 4 of post-natal UPJO-like HN, were shown to be independent risk factors for surgery (6, 23, 33, 34). In a prospective study including 501 UPJO-like HN patients, Braga et al. showed that the pyeloplasty rate in patients with SFU grades 3 and 4 was significantly higher than that in those with SFU grades I and II (2% vs. 32%) (23). In a meta-analysis, Lee et al. had demonstrated that severe hydronephrosis (antenatal APD > 15 mm) found during the third trimester had an 88% chance of post-natal pathology (35). Dias et al. had also established that with a prenatal APD > 18 mm in the third trimester and >16 mm in the postnatal period, the sensitivity and specificity of eventually needing pyeloplasty for UPJO-like HN were 100 and 86% (10). Renal length discrepancy on ultrasound has already been shown to be a significantly reliable predictor of abnormal DMSA scans, representing function, and SFU grade, representing obstructive severity. Khazaei et al. showed in children of all ages with a left kidney longer than the right by ≥10 mm or right longer than the left by ≥7 mm corresponded with a positive predictive value (PPV) of 79 and 100% of abnormal DMSA scan (36). Kelley et al. had found that an increase in renal length was significantly associated with SFU 3 and 4 as compared to SFU 1 and 2 (37). The three parameters chosen for the PPS have thus been shown to capture significant anatomical and functional measures independently, so the next logical step was to combine them into a single scoring system.

Though drop in differential renal function (DRF) is commonly listed as an indication for pyeloplasty, it has been omitted from the PPS formula. DRF can occasionally be misleading with the supra-normal differential renal function (SNDRF) phenomenon. A finding of SNDRF is generally defined as when the hydronephrotic kidney is found to have higher than normal DRF (>55%) (38, 39). It is hypothesized that this finding does not reflect true elevated function but reflects hyper-filtration in the setting of obstruction (38). SNDRF has been found in studies to be associated with significant post-operative decrease in DRF (38, 40). Pippi Salle et al. suggested that SNDRF observed during renography is a true phenomenon and that parenchymal proximity and distribution in relation to the pelvis are critical determinants, thus recommending the conjugate view technique for HN renography (41). There is intrinsic measurement error in renal scans of hydronephrotic kidneys making DRF measurement unreliable, due to variation in technique and the presence of the SNDRF phenomenon. Thus, DRF measurements do not have a consistent unidirectional relationship with disease severity that can be effectively utilized in a prediction model such as with the PPS.

### Limitations

The main limitation of this study is that despite including widely accepted parameters that vary according to the severity of UPJO-like HN as components of the PPS, surgery indications are operator-dependent. A surgeon can determine his or her own criteria for pyeloplasty with some degree of flexibility from guidelines. Thus, the PPS system should be adopted for research at other centers for evaluation of the external validity of its predictive abilities.

Another limitation of the present study is that there have been debates regarding using pyeloplasty as an outcome in single-center studies involving UPJO-like HN. Those that are against using surgery as an outcome argue that pyeloplasty is inherently a surgeon's threshold for surgery rather than an objective point of need for surgery. However, pyeloplasty is one of the few concrete outcomes that is available in the UPJO-like HN natural history. If pyeloplasty cannot be considered as an outcome, no other concrete objective outcomes are currently available, with the exception of renal function loss and symptoms. As previously discussed, waiting for renal function to deteriorate to indicate surgery with the hopes to regain what has already been lost seems counter-intuitive, especially when nephron preservation is the goal. Using an objective criterion for surgery such as DRF deterioration has its own problems. A recent study, which utilized DRF <40% as the main indication for pyeloplasty, regardless of HN grade and APD, showed a much higher febrile UTI rate of 12.5% for patients followed non-surgically, when compared to previous studies (24). This abnormally higher UTI rate seen, which can be considered as a true outlier, was most likely secondary to waiting too long for renal function loss to occur to intervene.

The PPS system was tested with a dataset from a single tertiary pediatric hospital. In order to further assess its external validity, it should be verified at other centers with prospectively collected data and larger sample sizes.

Despite these limitations, we propose that there is value in attempting to predict which UPJO-like HN patients will undergo pyeloplasty, using the PPS. We encourage that this scoring system be adopted at other centers to verify its findings, and to possibly establish an objective, simple, standard measure to quantitively compare thresholds for surgery between various pediatric urologists.

## Data Availability Statement

The data analyzed in this study is subject to the following licenses/restrictions: the authors are not allowed to share data outside their institution without a data sharing agreement. Requests to access these datasets should be directed to Melissa McGrath, mcgram2@mcmaster.ca.

## Ethics Statement

The studies involving human participants were reviewed and approved by Hamilton Integrated Research Ethics Board. No consent from the participant was required, as the study is part of a long term ongoing database.

## Author Contributions

LB theorized the presented idea. MM and BL developed the theory and performed the computations. LB and FF verified the analytical methods. BL, MM, FF, and LB contributed to interpretation of the results. BL wrote the manuscript in consultation with MM, FF, and LB. LB supervised the project. All authors provided critical feedback and helped design the research, analysis, manuscript, and figures.

## Conflict of Interest

The authors declare that the research was conducted in the absence of any commercial or financial relationships that could be construed as a potential conflict of interest.

## Abbreviations

UPJO-like: Ureteropelvic junction obstruction-like (isolated hydronephrosis)
PPS: Pyeloplasty Prediction Score
HN: Hydronephrosis
SFU: Society of Fetal Urology
APD: Anteroposterior diameter
IQR: Interquartile range
CI: Confidence interval
ROC: Receiver operating characteristic curve
LR: Likelihood ratio
PPV: Positive predictive value
HSS: Hydronephrosis Severity Score
DMSA: Dimercaptosuccinic acid
DRF: Differential renal function
SNDRF: Supra-normal differential renal function.

## References

[1] Capolicchio J-P Braga LH Szymanski KM. Canadian urological association/pediatric urologists of Canada guideline on the investigation and management of antenatally detected hydronephrosis. Can Urol Assoc J. (2018) 12:85–92. 10.5489/cuaj.5094 29319488 PMC5905549

[2] Thorup J Jokela R Cortes D Nielsen OH. The results of 15 years of consistent strategy in treating antenatally suspected pelvi-ureteric junction obstruction. BJU Int. (2003) 91:850–2. 10.1046/j.1464-410x.2003.04228.x 12780846

[3] de Waard D Dik P Lilien MR Kok ET de Jong TPVM. Hypertension is an indication for surgery in children with ureteropelvic junction obstruction. J Urol. (2008) 179:1976–9. 10.1016/j.juro.2008.01.058 18355868

[4] Shulkin BL Mandell GA Cooper JA Leonard JC Majd M Parisi MT. Procedure guideline for diuretic renography in children 3.0. J Nucl Med Technol. (2008) 36:162–8. 10.2967/jnmt.108.056622 18765635

[5] Braga LH McGrath M Farrokhyar F Jegatheeswaran K Lorenzo AJ Society for fetal urology classification vs urinary tract dilation grading system for prognostication in prenatal hydronephrosis: a time to resolution analysis. J Urol. (2018) 199:1615–21. 10.1016/j.juro.2017.11.077 29198999

[6] Fernbach SK Maizels M Conway JJ. Ultrasound grading of hydronephrosis: introduction to the system used by the society for fetal urology. Pediatr Radiol. (1993) 23:478–80. 10.1007/bf02012459 8255658

[7] Coplen DE Austin PF Yan Y Blanco VM Dicke JM. The magnitude of fetal renal pelvic dilatation can identify obstructive postnatal hydronephrosis, and direct postnatal evaluation and management. J Urol. (2006) 176:724–7. 10.1016/j.juro.2006.03.079 16813930

[8] Duin LK Willekes C Koster-Kamphuis L Offermans J Nijhuis JG. Fetal hydronephrosis: does adding an extra parameter improve detection of neonatal uropathies? J Matern Fetal Neonatal Med. (2012) 25:920–3. 10.3109/14767058.2011.600365 21843111

[9] Psooy K Pike J. Investigation and management of antenatally detected hydronephrosis. Can Urol Assoc J. (2009) 3:69–72. 10.5489/cuaj.1027 19293983 PMC2645869

[10] Dias CS Silva JMP Pereira AK Marino VS Silva LA Coelho AM. Diagnostic accuracy of renal pelvic dilatation for detecting surgically managed ureteropelvic junction obstruction. J Urol. (2013) 190:661–6. 10.1016/j.juro.2013.02.014 23416643

[11] Barbosa JABA Chow JS Benson CB Yorioka MA Bull AS Retik AB. Postnatal longitudinal evaluation of children diagnosed with prenatal hydronephrosis: insights in natural history and referral pattern. Prenat Diagn. (2012) 32:1242–9. 10.1002/pd.3989 23090854

[12] Dicke JM Blanco VM Yan Y Coplen DE. The type and frequency of fetal renal disorders and management of renal pelvis dilatation. J Ultrasound Med. (2006) 25:973–7. 10.7863/jum.2006.25.8.973 16870890

[13] Nguyen HT Benson CB Bromley B Campbell JB Chow J Coleman B. Multidisciplinary consensus on the classification of prenatal and postnatal urinary tract dilation (UTD classification system). J Pediatr Urol. (2014) 10:982–98. 10.1016/j.jpurol.2014.10.002 25435247

[14] Arora S Yadav P Kumar M Singh SK Sureka SK Mittal V. Predictors for the need of surgery in antenatally detected hydronephrosis due to UPJ obstruction–a prospective multivariate analysis. J Pediatr Urol. (2015) 11:248.e1–5. 10.1016/j.jpurol.2015.02.008 25986208

[15] Dhillon HK. Prenatally diagnosed hydronephrosis: the great ormond street experience. Br J Urol. (1998) 81(Suppl 2):39–44. 10.1046/j.1464-410x.1998.0810s2039.x 9602794

[16] Partin A Peters C Kavoussi L Dmochowski R Wein A Campbell-Walsh-Wein Urology, 12th Edition. Philadelphia, PA: Elsevier (2020).

[17] Castagnetti M Novara G Beniamin F Vezzu B Rigamonti W Artibani W. Scintigraphic renal function after unilateral pyeloplasty in children: a systematic review. BJU Int. (2008) 102:862–8. 10.1111/j.1464-410X.2008.07597.x 18336599

[18] Xu G Xu M Ma J Chen Z Jiang D Hong Z. An initial differential renal function between 35% and 40% has greater probability of leading to normal after pyeloplasty in patients with unilateral pelvic-ureteric junction obstruction. Int Urol Nephrol. (2017) 49:1701–6. 10.1007/s11255-017-1665-0 28795269

[19] Peters CA Urinary tract obstruction in children. J Urol. (1995) 154:1874 10.1016/s0022-5347(01)66815-0 7563375

[20] Koff SA Campbell KD. The nonoperative management of unilateral neonatal hydronephrosis: natural history of poorly functioning kidneys. J Urol. (1994) 152:593–5. 10.1016/s0022-5347(17)32658-7 8021976

[21] Koff SA. Neonatal management of unilateral hydronephrosis. Role for delayed intervention. Urol Clin North Am. (1998) 25:181–6. 10.1016/s0094-0143(05)70006-9 9633573

[22] Onen A Jayanthi VR Koff SA. Long-term followup of prenatally detected severe bilateral newborn hydronephrosis initially managed nonoperatively. J Urol. (2002) 168:1118–20. 10.1097/01.ju.0000024449.19337.8d 12187248

[23] Braga LH McGrath M Farrokhyar F Jegatheeswaran K Lorenzo AJ. Associations of initial society for fetal urology grades and urinary tract dilatation risk groups with clinical outcomes in patients with isolated prenatal hydronephrosis. J Urol. (2017) 197:831–7. 10.1016/j.juro.2016.08.099 27590478

[24] Alsabban A Romao R Dow T Anderson P MacLellan D Severe ureteropelvic junction obstruction (UPJO)-like hydronephrosis in asymptomatic infants: to operate or not? In: SPU 68th Annual Meeting (2020).

[25] Babu R Rathish VR Sai V. Functional outcomes of early versus delayed pyeloplasty in prenatally diagnosed pelvi-ureteric junction obstruction. J Pediatr Urol. (2015) 11:63.e1–5. 10.1016/j.jpurol.2014.10.007 25837703

[26] Tabari AK Atqiaee K Mohajerzadeh L Rouzrokh M Ghoroubi J Alam A Early pyeloplasty versus conservative management of severe ureteropelvic junction obstruction in asymptomatic infants. J Pediatr Surg. (2020) S0022–3468:30513–5. 10.1016/j.jpedsurg.2019.08.006 31495506

[27] Babu R Venkatachalapathy E Sai V. Hydronephrosis severity score: an objective assessment of hydronephrosis severity in children-a preliminary report. J Pediatr Urol. (2019) 15:68.e1–e6. 10.1016/j.jpurol.2018.09.020 30392886

[28] Piepsz A. Antenatal detection of pelviureteric junction stenosis: main controversies. Semin Nucl Med. (2011) 41:11–19. 10.1053/j.semnuclmed.2010.07.008 21111856

[29] Wong DC Rossleigh MA Farnsworth RH. Diuretic renography with the addition of quantitative gravity-assisted drainage in infants and children. J Nucl Med. (2000) 41:1030−6. 10855630

[30] Turkolmez S Atasever T Turkolmez K Gogus O. Comparison of three different diuretic renal scintigraphy protocols in patients with dilated upper urinary tracts. Clin Nucl Med. (2004) 29:154–60. 10.1097/01.rlu.0000113852.57445.23 15162983

[31] Kumar MT Hanuwant S Comparison of the F+20 and F-15 diuresis technetium-99m diethylenetriaminepentacetate renography protocols for diagnosis of ureteropelvic junction obstruction in adult patients with hydronephrosis. Indian J Nucl Med. (2018) 33:39–42. 10.4103/ijnm.IJNM_113_17 29430113 PMC5798096

[32] Donoso G Ham H Tondeur M Piepsz A. Influence of early furosemide injection on the split renal function. Nucl Med Commun. (2003) 24:791–5. 10.1097/01.mnm.0000080253.50447.94 12813198

[33] Chertin B Pollack A Koulikov D Rabinowitz R Hain D Hadas-Halpren I. Conservative treatment of ureteropelvic junction obstruction in children with antenatal diagnosis of hydronephrosis: lessons learned after 16 years of follow-up. Eur Urol. (2006) 49:734–8. 10.1016/j.eururo.2006.01.046 16504374

[34] Ross SS Kardos S Krill A Bourland J Sprague B Majd M. Observation of infants with SFU grades 3-4 hydronephrosis: worsening drainage with serial diuresis renography indicates surgical intervention and helps prevent loss of renal function. J Pediatr Urol. (2011) 7:266–71. 10.1016/j.jpurol.2011.03.001 21527234

[35] Lee RS Cendron M Kinnamon DD Nguyen HT. Antenatal hydronephrosis as a predictor of postnatal outcome: a meta-analysis. Pediatrics. (2006) 118:586–93. 10.1542/peds.2006-0120 16882811

[36] Khazaei MR Mackie F Rosenberg AR Kainer G. Renal length discrepancy by ultrasound is a reliable predictor of an abnormal DMSA scan in children. Pediatr Nephrol. (2008) 23:99–105. 10.1007/s00467-007-0637-5 17962982

[37] Kelley JC White JT Goetz JT Romero E Leslie JA Prieto JC. Sonographic renal parenchymal measurements for the evaluation and management of ureteropelvic junction obstruction in children. Front Pediatr. (2016) 4:42. 10.3389/fped.2016.00042 27200323 PMC4858526

[38] Rickard M Braga LH Gandhi S Oliveria J-P Demaria J Lorenzo AJ Comparative outcome analysis of children who underwent pyeloplasty for ureteropelvic junction obstruction associated with or without supranormal differential renal function. Urology. (2017) 99:210–4. 10.1016/j.urology.2016.07.016 27450350

[39] Inanir S Biyikli N Noshari O Caliskan B Tugtepe H Erdil TY. Contradictory supranormal function in hydronephrotic kidneys: fact or artifact on pediatric MAG-3 renal scans? Clin Nucl Med. (2005) 30:91–6. 10.1097/00003072-200502000-00004 15647673

[40] Cho SY Kim IS Lee S-B Choi H Park K. Nature and fate of supranormal differential renal function: lessons from long-term follow-up after pyeloplasty. Urology. (2013) 81:163–7. 10.1016/j.urology.2012.09.017 23200971

[41] Pippi Salle JL Cook A Papanikolaou F Bagli D Breen SL Charron M. The importance of obtaining conjugate views on renographic evaluation of large hydronephrotic kidneys: an *in vitro* and ex vivo analysis. J Urol. (2008) 180:1559–65. 10.1016/j.juro.2008.06.010 18710764

